# Ephedra Herb extract activates/desensitizes transient receptor potential vanilloid 1 and reduces capsaicin-induced pain

**DOI:** 10.1007/s11418-016-1034-9

**Published:** 2016-09-08

**Authors:** Shunsuke Nakamori, Jun Takahashi, Sumiko Hyuga, Toshiko Tanaka-Kagawa, Hideto Jinno, Masashi Hyuga, Takashi Hakamatsuka, Hiroshi Odaguchi, Yukihiro Goda, Toshihiko Hanawa, Yoshinori Kobayashi

**Affiliations:** 1 Department of Pharmacognosy, School of Pharmacy, Kitasato University, 5-9-1 Shirokane, Minato-ku, Tokyo, 108-8641 Japan; 2 Oriental Medicine Research Center, Kitasato University, 5-9-1, Shirokane, Minato-ku, Tokyo, 108-8642 Japan; 3 Department of Biochemical Toxicology, Yokohama University of Pharmacy, 601 Matano-cho, Totsuka-ku, Yokohama, 245-0066 Japan; 4 Faculty of Pharmacy, Meijo University, 150 Yagotoyama, Tempaku-ku, Nagoya, 468-8503 Japan; 5 National Institute of Health Sciences, 1-18-1 Kamiyoga, Setagaya-ku, Tokyo, 158-8501 Japan

**Keywords:** Ephedra Herb, TRPV1, Pain, Nociception, Analgesia, Capsaicin

## Abstract

Kampo medicines containing Ephedra Herb (EH) such as eppikajutsubuto and makyoyokukanto are used to treat myalgia, arthralgia, and rheumatism. The analgesic effects of these Kampo medicines are attributed to the anti-inflammatory action of EH. However, the molecular mechanism of the analgesic effect of EH remains to be clarified. In this study, the effects of EH extract (EHE) on transient receptor potential vanilloid 1 (TRPV1), a nonselective ligand-gated cation channel, which plays an essential role in nociception on sensory neurons, were investigated using mTRPV1/Flp-In293 cells (stable mouse TRPV1-expressing transfectants). Administration of EHE increased the intracellular Ca^2+^ concentration in these cells, which was inhibited by the TRPV1 antagonist, *N*-(4-tert-butylphenyl)-1,2-dihydro-4-(3-chloropyridine-2-yl) tetrahydropyrazine-1-carboxamide (BCTC), indicating that EHE activated TRPV1. Examination of EHE-induced nociceptive pain in vivo revealed that an intradermal (i.d.) injection of EHE into the hind paw of mice induced paw licking, a pain-related behavior, and that the extract increased paw licking times in a dose-dependent manner. The EHE-induced paw licking was also inhibited by BCTC. An i.d. injection of EHE 30 min before administration of capsaicin decreased capsaicin-induced paw licking times. Similarly, oral administration of the extract also suppressed capsaicin-induced paw licking, without affecting the physical performance of the mice. These results suggest that EHE suppresses capsaicin-induced paw licking by regulating TRPV1 activity. Thus, the antinociceptive effects of EHE seem to be produced by its direct action on sensory neurons through TRPV1.

**Electronic supplementary material:**

The online version of this article (doi:10.1007/s11418-016-1034-9) contains supplementary material, which is available to authorized users.

## Introduction

Ephedra Herb (EH) is defined as the terrestrial stem of *Ephedra sinica* Staf, *E. intermedia* Schrenk et C. A. Meyer, or *E. equisetina* Bunge (Ephedraceae) [1]. It is one of the most important crude drugs used in Japan and is a component of many Kampo formulae such as maoto, kakkonto, eppikajutsubuto, makyoyokukanto, and maobushisaishinto (http://kconsort.umin.jp/framepage.html) that have been used to treat rheumatism, myalgia, and arthralgia [2–4]. The analgesic actions of these Kampo medicines are thought to result from the anti-inflammatory properties of EH [3, 5, 6]. Recently, we demonstrated that EH extract (EHE) suppresses the late phase of formalin-induced pain [7], which is characterized as an inflammatory pain related to the release of chemical mediators such as histamine, serotonin, bradykinin (BK), and prostaglandins (PGs), and is suppressed by nonsteroidal anti-inflammatory drugs [8]. Thus, EH is thought to be involved in the regulation of inflammatory pain. However, the molecular mechanism of its analgesic effect remains to be clarified.

Transient receptor potential vanilloid 1 (TRPV1) is a nonselective ligand-gated cation channel expressed in primary sensory nerves [9]. It is an integrator of many noxious physical and chemical stimuli such as heat (>43 °C), proton, and capsaicin [9, 10], as well as endogenous lipids such as anandamide and 12-(S)-hydroperoxyeicosatetraenoic acid [11–13]. Upon the activation of TRPV1 by these stimuli, a variety of pro-algesic neuropeptides such as substance P and calcitonin gene-related peptide are released from the peripheral nerve terminals, and subsequently, a neurogenic inflammation is induced [14]. Furthermore, TRPV1 is sensitized by the stimulation of certain inflammatory mediators such as BK and PGs, and is transported to the plasma membrane by nerve growth factor stimulation, which indirectly modulates TRPV1 through its phosphorylation [14–16]. These inflammatory mediators reduce the temperature threshold for activation of TRPV1 from 43 to 35 °C, thus inducing inflammatory pain [10].

In this study, we assessed the effects of EHE on mouse TRPV1-expressing Flp-In293 cells and capsaicin-induced pain in vivo, to investigate whether EHE elicits a direct nociceptive action.

## Materials and methods

### Materials

EHE (Lot. 2091037010) was purchased from Tsumura & Co. (Tokyo, Japan). The ephedrine content of EHE was quantified by HPLC, and was approximately 2 % (Fig. S1). Capsaicin and *N*-(4-tert-butylphenyl)-1,2-dihydro-4-(3-chloropyridine-2-yl) tetrahydropyrazine-1-carboxamide (BCTC) were purchased from Funakoshi Co., Ltd. (Tokyo, Japan).

### Animals

Specific pathogen-free ddY mice (5-week-old, male) were purchased from Japan SLC, Inc. (Shizuoka, Japan). Prior to experimentation, the mice were acclimatized for 1 week at a temperature of 25 ± 2 °C, humidity of 50 ± 10 %, and a 12-h light/12-h dark cycle. All animal experiments were performed between 10:00 a.m. and 5:00 p.m. The protocol for animal experiments was approved by the Institutional Animal Care and Use Committee of Kitasato University, and was performed in accordance with the Kitasato University guidelines for animal care, handling, and termination, which are in line with the international and Japanese guidelines for animal care and welfare.

### Transfectant Flp-In293 cells

Flp-In293 cells, derived from the HEK293 cell line containing a stably integrated FRT site, were transfected using Lipofectamine (Thermo Fisher Scientific Inc., Waltham, MA, USA) with pOG44 vector (Thermo Fisher Scientific Inc.) and pEF5/FRT/V5-DEST vector (Thermo Fisher Scientific Inc.) harboring full-length mouse TRPV1 cDNA (GeneCopoeia Inc., Rockville, MD, USA). The cells were cultivated in hygromycin B (200 μg/ml) for 4 weeks, and stable mouse TRPV1-expressing transfectants (mTRPV1/Flp-In293 cells) were established. The expression levels of TRPV1 protein in mTRPV1/Flp-In293 and Flp-In293 cells were determined by Western blotting using anti-TRPV1 antibody (Santa Cruz Biotechnology, Inc., Dallas, TX, USA).

### Cell culture

The mTRPV1/Flp-In293 cells were cultured in Dulbecco’s modified Eagle medium (DMEM) supplemented with 10 % fetal bovine serum (FBS), 2 mM GlutaMAX, 0.1 mM MEM non-essential amino acid solution (MEM NEAA), 200 µg/ml hygromycin B, 100 U/ml penicillin, and 100 µg/ml streptomycin at 37 °C in 5 % CO_2_. Flp-In293 cells were cultured under the same conditions without hygromycin B. The reagents for cell culture were purchased from Thermo Fisher Scientific Inc.

### Measurement of intracellular Ca^2+^ concentration in mTRPV1/Flp-In293 and Flp-In293 cells

Measurement of intracellular Ca^2+^ concentration was performed as previously described [17, 18]. Mouse TRPV1/Flp-In293 and Flp-In293 cells (4 × 10^4^ cells/well) were cultured in 100 µl of DMEM with 10 % FBS, 2 mM GlutaMAX, and 0.1 mM MEM NEAA in 96-well, poly-d-lysine black-walled, clear-bottomed plates (Greiner Bio-One, Frickenhausen, Germany) for 24 h. The medium was exchanged and the cells incubated in Hank’s balanced salt solution (HBSS) buffer and 20 mM HEPES buffer (pH 7.4) containing FLIPR^®^ calcium 5 assay reagent (Molecular Devices, Sunnyvale, CA, USA) for 1 h at 37 °C. The fluorescence was immediately measured using a FlexStation 3 microplate reader (Molecular Devices) (excitation at 485 nm and emission at 525 nm, using a 515-nm cut-off) for 20 s. Subsequently, HBSS buffer containing 0–1000 µg/ml of EHE or 0–0.2 µM capsaicin was added, and the fluorescence was immediately measured.

To examine the effect of BCTC, a TRPV1 antagonist, on EHE- or capsaicin-induced increase in intracellular Ca^2+^ concentration, HBSS buffer containing 0–10 nM BCTC was added after the initial fluorescence measurement was made. After 60 s, HBSS buffer containing 1000 µg/ml EHE or 0.00625 µM capsaicin was added, and the fluorescence was immediately measured. In these experiments, capsaicin and BCTC were dissolved in dimethyl sulfoxide (DMSO) and diluted with HBSS buffer. The final DMSO concentration was adjusted to within 0.1–0.2 %. The data were analyzed by Soft Max Pro 5.4 software (Molecular Devices).

### EHE- or capsaicin-induced paw licking test

The capsaicin-induced paw licking test was performed as previously described [19]. Mice were grouped into 4 or 5 groups treated with different doses of capsaicin or EHE, with 5–8 mice in each group. The mice were placed individually for adaptation in transparent acrylic cylinder cages with a height of 200 mm and a diameter of 100 mm. After 20 min, the mice were injected with 10 μl of vehicle (DMSO:Tween-80:saline = 1:1:8) containing 0.031–3.1 μg/paw capsaicin, or 0.3–10 mg/paw EHE, into the plantar surface of the left hind paw using a microsyringe (MS-NG50; Ito Microsyringe Co., Ltd, Tokyo, Japan) with a sharp-edged needle (28 G; Ito Microsyringe, Co., Ltd). The licking behavior was recorded using a digital video camera for a period of 5 min.

To investigate the effect of BCTC on EHE- or capsaicin-induced pain, the mice were grouped into 3 or 4 groups treated with three different doses of BCTC, with 3–7 mice in each group. The experiment was performed as described above except by injecting the mice with 10 μl of the vehicle containing 5 mg/paw EHE, together with 0.037–0.37 μg/paw BCTC.

To compare the time course of EHE-induced paw licking with that induced by capsaicin, the mice were grouped into 3 groups treated with vehicle, capsaicin, and EHE, with 3 mice in each group. The mice were injected with 10 µl of the vehicle, with or without 3 mg/paw EHE or 0.92 μg/paw capsaicin, into the plantar surface of the left hind paw. The licking behavior was recorded using the digital video camera for a period of 60 min.

### Analgesic effect of i.d. administration of EHE

The mice were grouped into 6 groups treated with vehicle, EHE or capsaicin, with 6 mice in each group. The mice were injected with 10 µl of the vehicle, with or without 3 mg/paw EHE or 0.92 μg/paw capsaicin into the plantar surface of the left hind paw. After 30 or 60 min, 0.18 μg/paw capsaicin (10 µl) was injected into the same area, and the licking time was measured.

### Analgesic effect of oral (p.o.) administration of EHE

The mice were grouped into 14 groups treated with either EHE or water, with 4–8 mice in each group. The mice were administered p.o. with 700 mg/kg of EHE. After a period of 0, 0.25, 0.5, 1, 2, 6, and 24 h, 0.18 μg/paw capsaicin (10 µl) was injected into the plantar surface of the left hind paw, and the licking time was measured for 5 min. To analyze the effects of different doses of EHE, the mice were administered p.o. with 87.5–700 mg/kg of EHE. After 30 min, 0.18 μg/paw capsaicin (10 µl) was injected as described above.

### Rotarod test

The rotarod test was performed as previously reported [20]. A rotarod treadmill (MK-600; Muromachi Kikai Co., Ltd, Tokyo, Japan) was used in this study. To adapt the mice to the rotarod, they were placed on the rod rotating at 28 rpm for 5 min each hour six times, 1 day before the test. On the day of the test, another episode of training was performed, and the mice that fell off the rotating shaft were excluded from the experiment. The mice were administered water or 175–700 mg/kg of EHE orally. After 30 min, we measured the endurance time the mice could remain on the rotarod.

### Statistical analysis

All data are expressed as mean ± standard error of the mean (SEM) and analyzed by one-way analysis of variance (ANOVA). Significant differences between the control and treatment groups were determined by Dunnett’s multiple comparison test or Student’s *t* test. All statistical analyses were performed using Prism 5 (GraphPad Software Inc., San Diego, CA, USA). Statistical significance was determined based on values of *p* < 0.05, 0.01, and 0.001.

## Results

### Confirmation of functional expression of mTRPV1 in mTRPV1/Flp-In293 cells

**Fig. 1:**
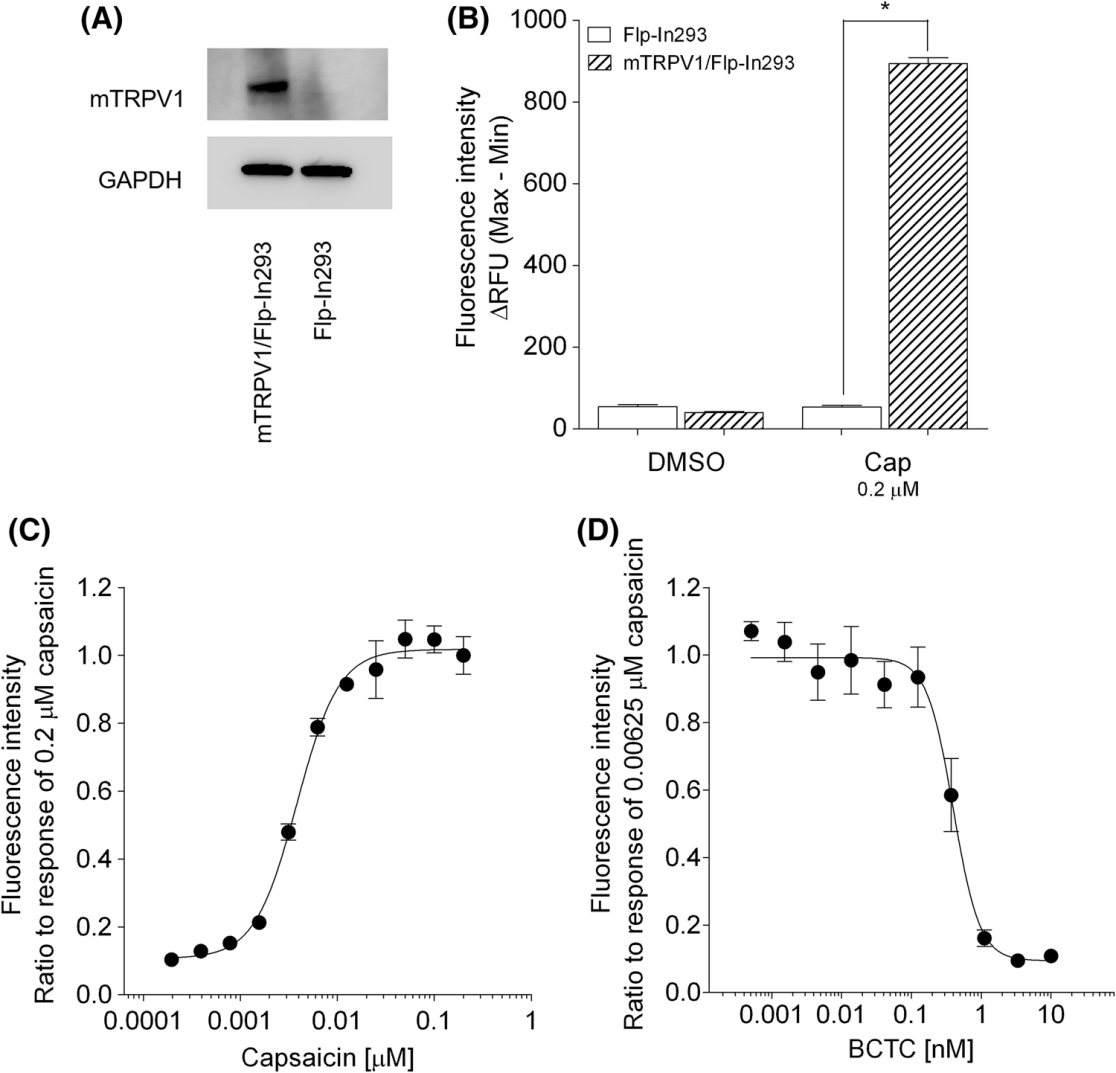
Confirmation of functional expression of mTRPV1 in mTRPV1/Flp-In293 cells. **a** Expression levels of mTRPV1 in mTRPV1/Flp-In293 and Flp-In293 cells were determined by Western blot analysis. **b** The effect of 0.2 μM capsaicin (Cap) on the uptake of Ca^2+^ in mTRPV1/Flp-In293 and Flp-In293 cells. **c** The ratio of fluorescence intensity induced by different concentrations of capsaicin over that induced by 0.2 µM capsaicin. **d** The ratio of fluorescence intensity induced by 0.00625 µM capsaicin in the presence of 0−10 nM BCTC over that induced in its absence. Each assay was performed in triplicate. The *error bar* represents the standard error. Statistical significance was determined with Tukey’s test; *p < 0.001 vs Flp-In293 cells

Expression levels of mTRPV1 were determined by Western blotting (Fig. 1a). Mouse TRPV1 was detected in mTRPV1/Flp-In293 cells, but not in Flp-In293 cells. The intracellular Ca^2+^ concentration in mTRPV1/Flp-In293 cells was increased by capsaicin, but that in Flp-In293 cells was unaffected (Fig. 1b). In addition, capsaicin produced a dose-dependent increase in intracellular Ca^2+^ concentration in mTRPV1/Flp-In293 cells (Fig. 1c). A capsaicin concentration of 0.00625 μM, which induced approximately 80 % of the maximum response produced by 0.2 μM capsaicin, was used to investigate the inhibitory effects of the TRPV1 antagonist, BCTC. As shown in Fig. 1d, BCTC dose-dependently inhibited the 0.00625 μM capsaicin-induced increase in intracellular Ca^2+^ concentration. These results indicated that mTRPV1 was functionally expressed in mTRPV1/Flp-In293 cells.

### Effect of EHE on TRPV1 studied using mTRPV1/Flp-In293 cells

**Fig. 2:**
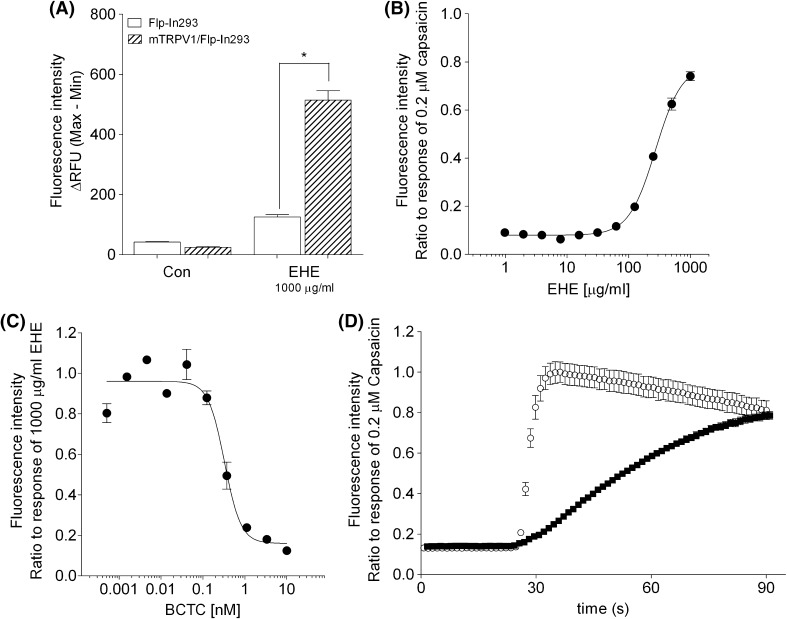
Effect of EHE on the uptake of Ca^2+^ into mTRPV1/Flp-In293 cells. **a** The effects of EHE on mTRPV1/Flp-In293 and Flp-In293 cells. **b** The ratio of fluorescence intensity induced by 0–1000 µg/ml EHE over that induced by 0.2 µM capsaicin. **c** The ratio of fluorescence intensity induced by 1000 µg/ml EHE in the presence of 0–10 nM BCTC over that induced in its absence. **d** Fluorescence kinetics measuring Ca^2+^ uptake by mTRPV1/Flp-In293 cells induced by 1000 µg/ml EHE (*closed circle*) and 0.2 μM capsaicin (*open circle*). Each assay was performed in triplicate. The *error bar* represents the standard error. Statistical significance was determined with Tukey’s test; *p < 0.001 vs Flp-In293 cells

The presence of EHE (1000 μg/ml) significantly increased the intracellular Ca^2+^ concentration in mTRPV1/Flp-In293 cells, but not in Flp-In293 cells (Fig. 2a). In addition, the increase in intracellular Ca^2+^ concentration by EHE was dose-dependent, with an EC_50_ value of 271.6 µg/ml (Fig. 2b), and was inhibited by BCTC in a similar manner (Fig. 2c). These results suggested that EHE directly activates mTRPV1. The time course of increasing intracellular Ca^2+^ concentration in mTRPV1/Flp-In293 cells induced by 1000 µg/ml EHE and 0.2 μM capsaicin is shown in Fig. 2d. The rate of increase of intracellular Ca^2+^ concentration in the presence of EHE was much slower than in the presence of capsaicin.

### EHE-induced nociceptive pain through the stimulation of TRPV1

**Fig. 3:**
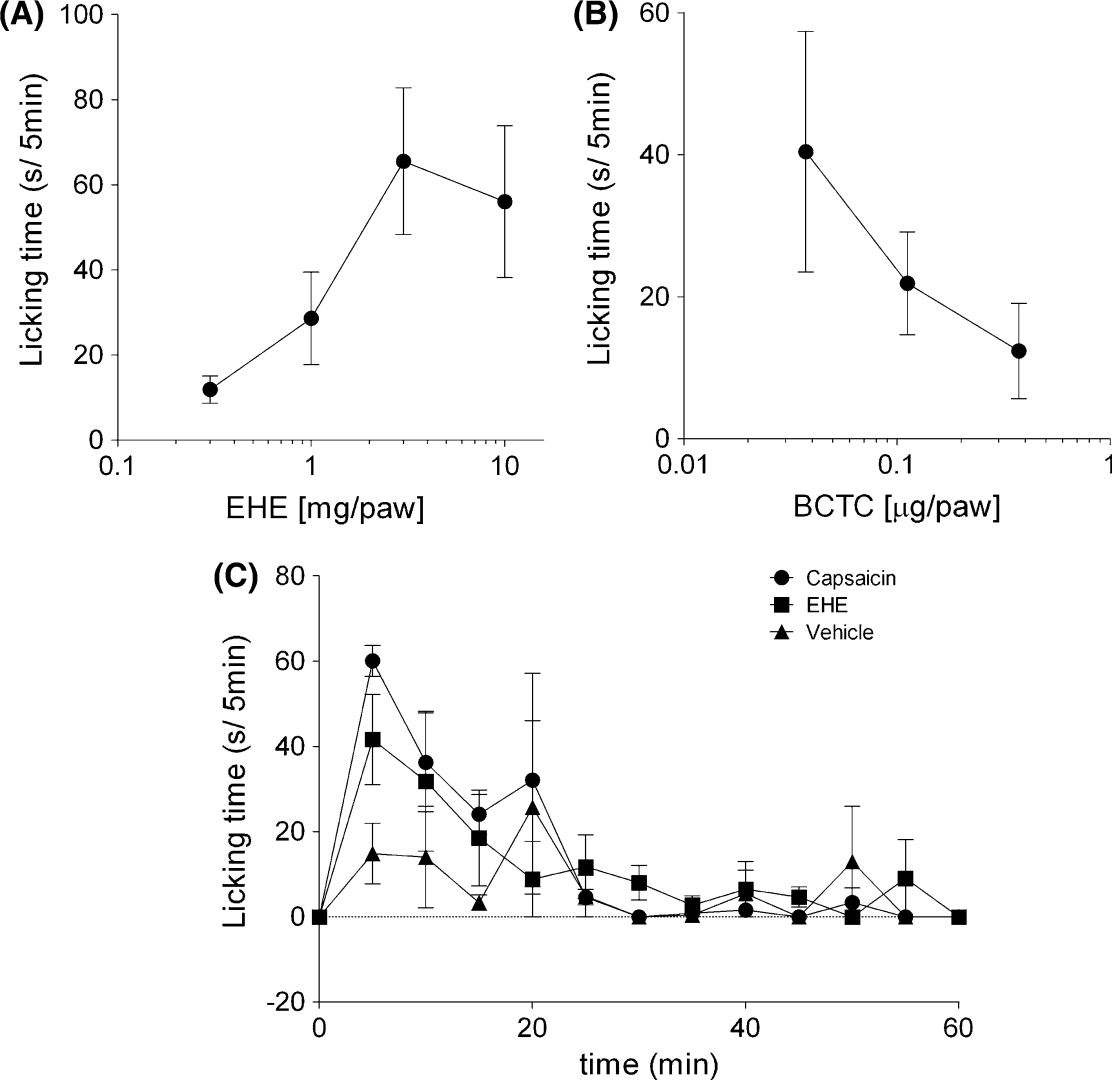
EHE-induced nociceptive pain. Mice were injected with 10 µl of vehicle (DMSO:Tween-80:physiological saline = 1:1:8) containing **a** 0.3–10 mg/paw EHE, **b** 5 mg/paw EHE with 0.037–0.37 μg/paw BCTC, and **c** 3 mg/paw EHE or 0.92 μg/paw capsaicin into the plantar surface of the* left* hind paw. Licking behaviors were observed for **a**, **b** 5 min, and **c** 60 min.* Data* represent the mean ± standard error of **a** 7–8, **b** 3–4, and **c** 3 mice

An i.d. injection of 0.3–10 mg/paw EHE (10 μl) into the hind paw of mice induced paw licking, a pain-related behavior, and the paw licking times increased in a dose-dependent manner (Fig. 3a). Similarly, the paw licking times decreased in a dose-dependent manner upon co-injection with BCTC (Fig. 3b), suggesting that EHE induces nociceptive pain through the activation of TRPV1. It is well known that capsaicin induces transient paw licking. The time course of EHE-induced paw licking was similar to that induced by capsaicin. The paw licking behavior induced by capsaicin and EHE was not observed starting from 25 min after the injection (Fig. 3c).

### Desensitization of TRPV1 in peripheral sensory nerves by EHE

**Fig. 4:**
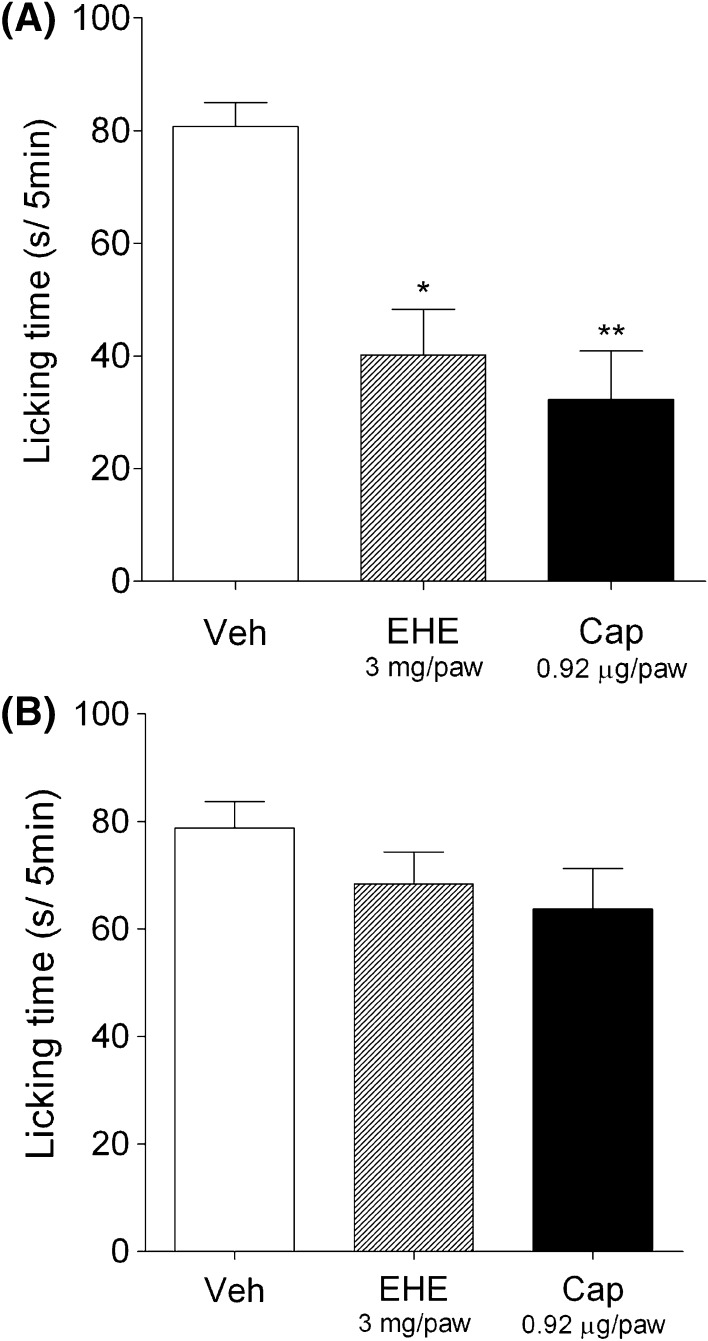
Suppression of capsaicin-induced pain by i.d. administration of EHE. Mice were injected with 10 µl of vehicle (DMSO:Tween-80:physiological saline = 1:1:8) containing 3 mg/paw EHE or 0.92 μg/paw capsaicin (Cap) into the plantar surface of* left* hind paw. After 30 min (**a**) or 60 min (**b**), 0.18 μg/paw capsaicin was injected into the same area. Licking behaviors were observed for 5 min.* Data* represent the mean ± standard error of 6 mice. Statistical significance was determined with Dunnett’s test; *p < 0.01, and **p < 0.001 vs vehicle group (Veh)

It is well known that a large dose of capsaicin desensitizes TRPV1, relieving nociceptive pain. In this experiment, the transient desensitization of capsaicin-induced pain was triggered by i.d. administration of a single large dose of EHE. Capsaicin-induced paw licking time significantly decreased 30 min after the injection of 3 mg/paw EHE or 0.92 μg/paw capsaicin into the hind paw (Fig. 4a); the desensitizing effects were abolished 60 min after their injection (Fig. 4b).

### Analgesic effect of oral administration of EHE on capsaicin-induced nociceptive pain

**Fig. 5:**
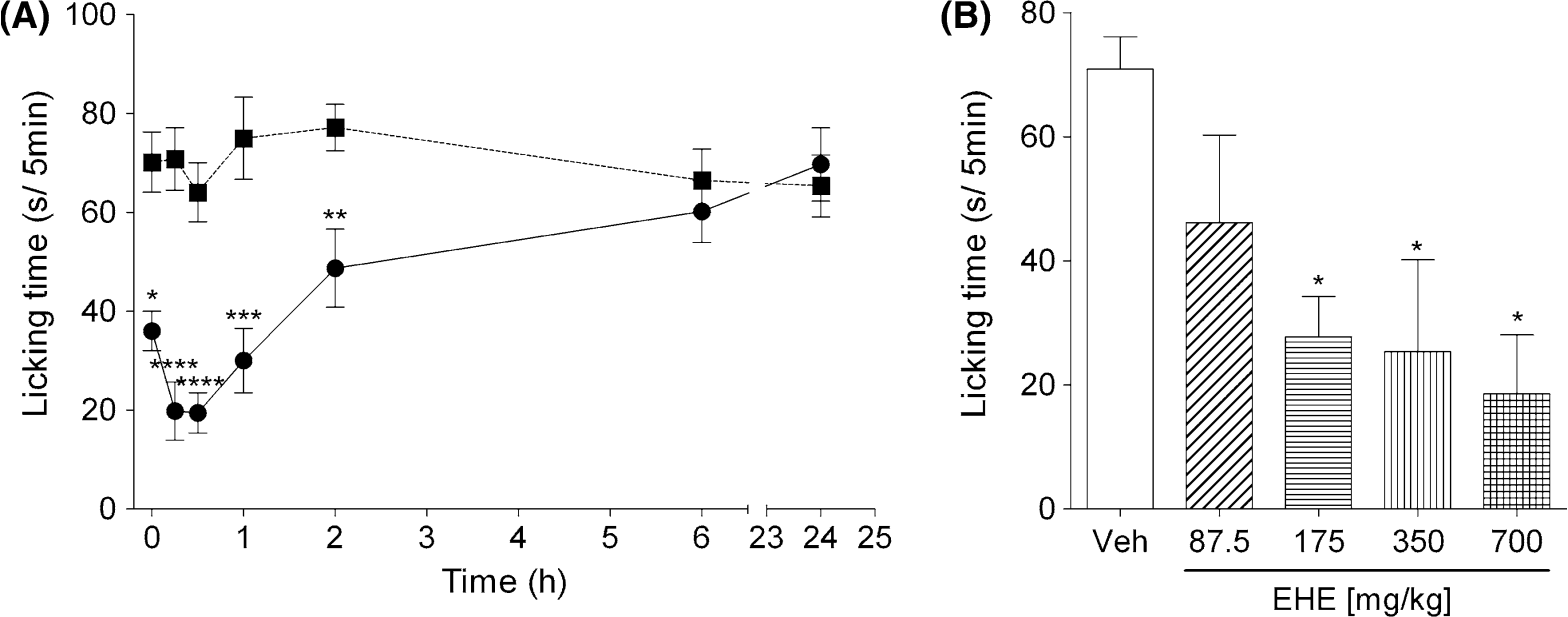
Suppression of capsaicin-induced pain by p.o. administration of EHE. **a** Mice were orally administered 700 mg/kg of EHE (*closed circle*) or water (*closed square*). After 0, 0.25, 0.5, 1, 2, 6, and 24 h, the mice were injected with 10 µl of solution (DMSO:Tween-80:physiological saline = 1:1:8) containing 0.06 mM capsaicin into the plantar surface of left hind paw. **b** Mice were orally administered 87.5–700 mg/kg of EHE. After 30 min, the mice were injected with 10 µl of solution (DMSO:Tween-80:physiological saline = 1:1:8) containing 0.18 μg/paw capsaicin into the plantar surface of* left* hind paw. Licking behaviors were observed for 5 min.* Data* represent the mean ± standard error of **a** 4–8 and **b** 3–4 mice. Statistical significance was determined with **a** Student’s *t* test; *p < 0.05, **p < 0.01, ***p < 0.001, and ****p < 0.0001 vs the water group and **b** Dunnett’s test; *p < 0.05 vs vehicle group (Veh)

**Fig. 6:**
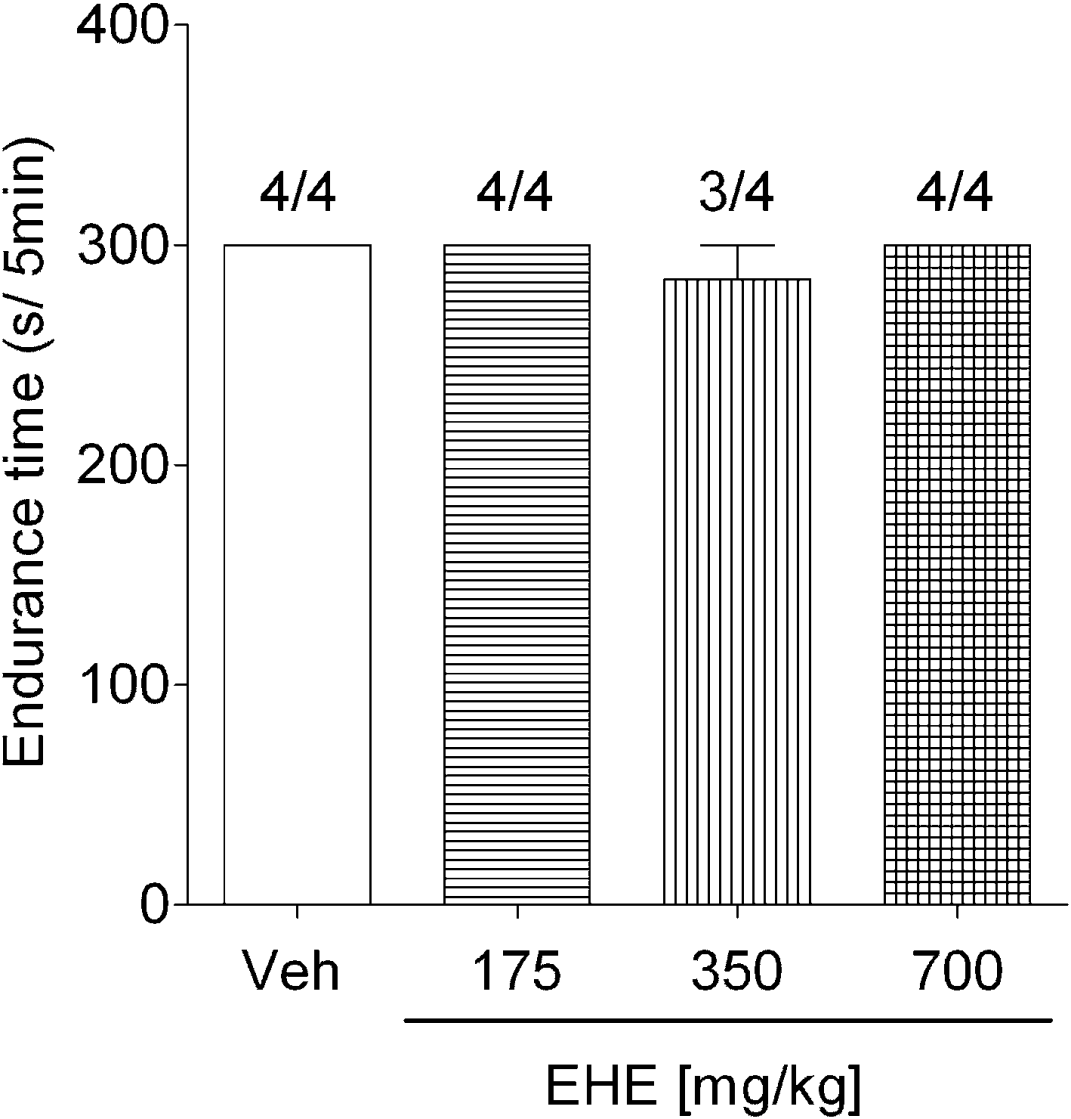
Effect of EHE on rotarod performance of mice. Mice were orally administered 175–700 mg/kg of EHE. After 30 min, the mice were placed on the rod rotating at 28 rpm, and their performance was observed for 5 min.* Data* represent the mean ± standard error of 4 mice.* Values* above the* columns* represent the number of mice that did not fall during a period of 5min. Statistical significance was determined with Dunnett’s test; *p < 0.05 vs vehicle group (Veh)

Capsaicin-induced paw licking times were recorded between 0 and 24 h after p.o. administration of EHE (700 mg/kg). Paw licking times significantly decreased for up to 2 h after the administration of EHE, with the maximal effect observed between 15 and 30 min after EHE administration (Fig. 5a). The antinociceptive effect of p.o. administration of EHE reduced the capsaicin-induced paw licking times in a dose-dependent manner (Fig. 5b). Furthermore, the physical performance of mice was evaluated 30 min after p.o. administration of EHE (175–700 mg/kg) and compared with mice from the vehicle group using a rotarod treadmill. There were no significant differences in physical performance between the two groups (Fig. 6). Thus, these results indicated that p.o. administration of EHE has an analgesic effect on capsaicin-induced pain.

## Discussion

The analgesic effect of EH has previously been thought to be an indirect effect elicited through anti-inflammatory action [5, 6]. In this study, we demonstrated that EHE exhibits a direct antinociceptive effect by affecting the TRPV1-mediated nociceptive pathway. TRPV1 is a polymodal receptor that converts multiple noxious stimulation into electric signals [10]. It is extensively modulated by neurotransmitters, inflammatory cytokines, growth factors, local hormones, and oxidative chemicals, thereby serving as an integrator for processing nociceptive information [21].

We investigated the effect of EHE on TRPV1 using stable mouse TRPV1-expressing transfected mTRPV1/Flp-In293 cells (Fig. 2), and showed that EHE increased the intracellular Ca^2+^ concentration in these cells, and that this increase was inhibited by BCTC, a TRPV1 antagonist (Fig. 2a–c) [22–25]. These results indicate the presence of components in EHE that directly activate mTRPV1. The kinetics of the increase in the Ca^2+^ concentration in mTRPV1/Flp-In293 cells induced by EHE was different from that produced by capsaicin (Fig. 2d), suggesting that the binding mode of the compounds in EHE to TRPV1 might be different from that of capsaicin. A number of TRPV1 activators have been found in natural products, including capsaicin, 6-gingerol, piperine, and evodiamine found in hot pepper, ginger, black pepper, and evodia fruits, respectively [26, 27]. However, there are currently no reports describing the effect of the compounds contained in EHE on TRPV1. Investigations are now underway to identify the active molecules in EHE.

Capsaicin, a TRPV1 ligand, is thought to stimulate nociceptive pain by the activation of TRPV1 on sensory neurons, since the nociceptive pain that it induces is specifically inhibited by BCTC (Fig. S2) [23]. An i.d. injection of EHE induced paw licking in mice that was suppressed by BCTC administration (Fig. 3), suggesting that EHE similarly induces pain through the activation of TRPV1 on sensory neurons in vivo. Moreover, pretreatment with a high dose of EHE suppressed the capsaicin-induced paw licking, suggesting that EHE elicits an analgesic action by affecting TRPV1 function on peripheral sensory nerves. Although capsaicin is known to induce pain, it also exhibits analgesic effects [28]. Therefore, capsaicin has been used to treat neuropathic pain, and there are many clinical trials testing its topical application, such as the use of capsaicin patches [28–30]. The analgesic effects of capsaicin are considered to be derived from both the desensitization of TRPV1 and defunctionalization of TRPV1-positive nerves [16]. The desensitization of TRPV1 by capsaicin depends on an increase in intracellular Ca^2+^ concentration [31–33], and the analgesic effects induced by this process are thought to be transient. Several mechanisms of desensitization of TRPV1 have been suggested—(1) the phosphatase, calcineurin, is activated by an increase in intracellular Ca^2+^ concentration through the activation of TRPV1, and the dephosphorylation of TRPV1 by calcineurin is followed by the desensitization of TRPV1 while it recovers from desensitization through the inhibition of calcineurin [34]; (2) the interaction between the Ca^2+^/calmodulin complex and TRPV1 induces a reduction in TRPV1 responsiveness [35–37]; and (3) TRPV1 agonists that rapidly downregulate the membrane expression of TRPV1 through endocytosis and lysosomal degradation. This process is modulated by an increase in intracellular Ca^2+^ concentration and protein kinase A-dependent phosphorylation of TRPV1 [38]. In contrast, the long-lasting analgesic effect of capsaicin is considered to arise from the defunctionalization of TRPV1-positive nerves [28–30]. Prolonged activation of TRPV1 by repeated or high-concentration capsaicin treatment causes long-lasting loss of sensitivity of sensory neurons, as the overload of Ca^2+^ by prolonged activation of TRPV1 induces degeneration of sensory nerve terminals [39].

In this study, the analgesic effect of an i.d. injection of a high dose of EHE (3 mg/paw) or capsaicin (0.92 μg/paw) was transient, and was abolished 60 min after the injection of EHE or capsaicin (Fig. 4). Therefore, this analgesic effect possibly originated from the desensitization of TRPV1. Oral administration of EHE also relieved capsaicin-induced paw licking without a drop in physical performance. Surprisingly, the analgesic effect of EHE was evident immediately after its p.o. administration, and was highest between 15 and 30 min after administration (Fig. 5a). These results suggest that EHE contains components that are rapidly absorbed from the digestive system. Wei et al. [40] recently reported that ephedrine alkaloids, the major alkaloid components of EH, are rapidly absorbed in rats. However, ephedrine did not activate or inhibit TRPV1 (Fig. S3A and B). Efforts are underway in our laboratory to identify the active compounds of EHE.

Desensitization of TRPV1 is likely to be one of the molecular mechanisms of EHE action following its i.d. administration. However, it is unclear if the analgesic effect of EHE by oral administration (Fig. 5a) is similarly mediated by the TRPV1 pathway. Thus, further investigation of the mechanism of the analgesic effect of oral administration of EHE is required.

Antagonists of TRPV1 have been reported to alleviate various types of pain, such as neuropathic pain, inflammatory pain, and allodynia [41]. However, these agents also induce undesirable side-effects such as hyperthermia and an increase in the threshold of noxious heat in humans [42, 43]. On the other hand, EH has not been reported to induce the above adverse effects. Therefore, it is possible that EHE is more effective than other TRPV1 antagonists in addressing acute and chronic pain.

We report for the first time that EHE activates TRPV1 in vitro. Topical administration of EHE activates and desensitizes TRPV1, and alleviates capsaicin-induced pain in vivo. These results suggest that the TRPV1 pathway may be integral to the molecular mechanism of EHE action. Although oral administration of EHE also reduced the capsaicin-induced pain, the mechanism of action remains to be elucidated.

## Electronic supplementary material

Below is the link to the electronic supplementary material.

**Fig. S1** Chromatogram of EHE obtained using an HPLC system (Shimadzu, Kyoto, Japan) consisting of an SIL-20A auto-injector, SPD-M20A photodiode array detector, LC-20AD pump, DGU-20A3 degasser, and CBM-20A communications bus module. Separations were carried out with an YMC-Triart C18 plus column (5 µm particle size, 4.6 mm (inner diameter) × 150 mm; YMC Co., Ltd, Kyoto, Japan). The mobile phase was a mixture of water, acetonitrile, and phosphoric acid (650:350:1, v/v/v) containing 0.5 % SDS delivered at a flow rate of 1 ml/min. The column temperature was maintained at 40 °C with a CTO-20A column oven (Shimadzu). The detection wavelength was set at 210 nm for quantitative determination. The test samples were resolved by methanol and injected (10 µl injection volume) by an auto-injector. (TIFF 112 kb)

**Fig. S2** **a** Capsaicin-induced nociceptive pain. Mice were injected with 10 μl of solution (DMSO:Tween-80:physiological saline = 1:1:8) containing 0.031–3.1 μg/paw capsaicin into the plantar surface of the *left* hind paw using a microsyringe. Licking behaviors were observed for 5 min. Each data represents the mean ± standard error of 5–7 mice. (TIFF 37 kb)

**b** Inhibition of the capsaicin-induced pain by BCTC. Mice were injected with 10 μl of solution (DMSO:Tween-80:physiological saline = 1:1:8) containing 0.18 μg/paw capsaicin with 0.0011–0.037 μg/paw BCTC into the plantar surface of the *left* hind paw using a microsyringe. Licking behaviors were observed for 5 min. Each data represents the mean ± standard error of 6 mice. (TIFF 34 kb)

**Fig. S3** **a** Effect of ephedrine on the uptake of Ca^2+^ into mTRPV1/Flp-In293 cells through the activation of TRPV1. The ratio of fluorescence intensity induced by HBSS buffer alone (Con) and buffer containing 0.2 µM capsaicin (Cap), 1000 µg/ml EHE, or 40 µg/ml ephedrine (Eph), in the absence and presence of 1 µM BCTC, over that induced by 0.2 µM capsaicin. (TIFF 61 kb)

**b** Effects of ephedrine on the uptake of Ca^2+^ into mTRPV1/Flp-In293 cells through TRPV1 activation by capsaicin. The ratio of fluorescence intensity induced by 0−0.2 µM capsaicin in the absence (*closed circle*) and presence of 40 μg/ml ephedrine (*closed square*) or 1 nM BCTC (*closed triangle*), over that induced by 0.2 μM capsaicin. Each assay was performed in triplicate. The error bar represents the standard error. Statistical significance was determined with Tukey’s test; *p < 0.001 vs BCTC-treated group. (TIFF 60 kb)

## References

[1] Ministry of Health, Labor and Welfare of Japan (2010) The Japanese pharmacopoeia, 16th edn. Tokyo

[2] Urabe A, Shimada K, Kwai S (2015). Today’s drug therapy in 2015.

[3] Toriizuka K (2003). Monographs of pharmacological research on traditional herbal medicines (Shoyaku no yakuso yakuri).

[4] Batz F, Hitchens K, Jellin JM (2012). Pharmacist’s letter/Prescriber’s letter natural medicines comprehensive database.

[5] Yeom MJ, Lee HC, Kim GH, Lee HJ, Shim I, Oh SK, Kang SK, Hahm DH (2006). Anti-arthritic effects of Ephedra sinica STAPF herb-acupuncture: inhibition of lipopolysaccharide-induced inflammation and adjuvant-induced polyarthritis. J Pharmacol Sci.

[6] Kasahara Y, Hikino H, Tsurufuji S, Watanabe M, Ohuchi K (1985). Antiinflammatory actions of ephedrines in acute inflammations. Planta Med.

[7] Hyuga S, Hyuga M, Oshima M, Maruyama M, Kamakura H, Yamashita T, Yoshimura M, Amakura Y, Hakamatsuka T, Odaguchi H, Goda Y, Hanawa T (2016). Ephedrine alkaloids-free Ephedra Herb extract: a safer alternative to ephedra with comparable analgesic, anticancer, and anti-influenza activities. J Nat Med.

[8] Barrot M (2012). Tests and models of nociception and pain in rodents. Neuroscience.

[9] Caterina MJ, Schumacher MA, Tominaga M, Rosen TA, Levine JD, Julius D (1997). The capsaicin receptor: a heat-activated ion channel in the pain pathway. Nature.

[10] Tominaga M, Caterina MJ, Malmberg AB, Rosen TA, Gilbert H, Skinner K, Raumann BE, Basbaum AI, Julius D (1998). The cloned capsaicin receptor integrates multiple pain-producing stimuli. Neuron.

[11] Zygmunt PM, Petersson J, Andersson DA, Chuang H, Sørgård M, Di Marzo V, Julius D, Högestätt ED (1999). Vanilloid receptors on sensory nerves mediate the vasodilator action of anandamide. Nature.

[12] Hwang SW, Cho H, Kwak J, Lee SY, Kang CJ, Jung J, Cho S, Min KH, Suh YG, Kim D, Oh U (2000). Direct activation of capsaicin receptors by products of lipoxygenases: endogenous capsaicin-like substances. Proc Natl Acad Sci USA.

[13] Szolcsányi J, Sándor Z (2012). Multisteric TRPV1 nocisensor: a target for analgesics. Trends Pharmacol Sci.

[14] Planells-Cases R, Garcìa-Sanz N, Morenilla-Palao C, Ferrer-Montiel A (2005). Functional aspects and mechanisms of TRPV1 involvement in neurogenic inflammation that leads to thermal hyperalgesia. Pflug Arch.

[15] Lewin GR, Nykjaer A (2014). Pro-neurotrophins, sortilin, and nociception. Eur J Neurosci.

[16] O’Neill J, Brock C, Olesen AE, Andresen T, Nilsson M, Dickenson AH (2012). Unravelling the mystery of capsaicin: a tool to understand and treat pain. Pharmacol Rev.

[17] Ohkawara S, Tanaka-Kagawa T, Furukawa Y, Nishimura T, Jinno H (2010). Activation of the human transient receptor potential vanilloid subtype 1 by essential oils. Biol Pharm Bull.

[18] Ohkawara S, Tanaka-Kagawa T, Furukawa Y, Jinno H (2012). Methylglyoxal activates the human transient receptor potential ankyrin 1 channel. J Toxicol Sci.

[19] Melo CM, Maia JL, Cavalcante IJ, Lima MA, Vieira GA, Silveira ER, Rao VS, Santos FA (2006). 12-Acetoxyhawtriwaic acid lactone, a diterpene from *Egletes viscosa*, attenuates capsaicin-induced ear edema and hindpaw nociception in mice: possible mechanisms. Planta Med.

[20] Rosland JH, Hunskaar S, Hole K (1990). Diazepam attenuates morphine antinociception test-dependently in mice. Pharmacol Toxicol.

[21] Wang S, Chuang HH (2011). C-terminal dimerization activates the nociceptive transduction channel transient receptor potential vanilloid 1. J Biol Chem.

[22] Valenzano KJ, Grant ER, Wu G, Hachicha M, Schmid L, Tafesse L, Sun Q, Rotshteyn Y, Francis J, Limberis J, Malik S, Whittemore ER, Hodges D (2003). N-(4-Tertiarybutylphenyl)-4-(3-chloropyridin-2-yl)tetrahydropyrazine-1(2H)-carbox-amide (BCTC), a novel, orally effective vanilloid receptor 1 antagonist with analgesic properties: I. in vitro characterization and pharmacokinetic properties. J Pharmacol Exp Ther.

[23] Pomonis JD, Harrison JE, Mark L, Bristol DR, Valenzano KJ, Walker K (2003). N-(4-Tertiarybutylphenyl)-4-(3-chloropyridin-2-yl)tetrahydropyrazine-1(2H)-carbox-amide (BCTC), a novel, orally effective vanilloid receptor 1 antagonist with analgesic properties: II. in vivo characterization in rat models of inflammatory and neuropathic pain. J Pharmacol Exp Ther.

[24] Okumi H, Takashima K, Matsumoto K, Namiki T, Terasawa K, Horie S (2012). Dietary agonists of TRPV1 inhibit gastric acid secretion in mice. Planta Med.

[25] Gavva NR, Tamir R, Klionsky L, Norman MH, Louis JC, Wild KD, Treanor JJ (2005). Proton activation does not alter antagonist interaction with the capsaicin-binding pocket of TRPV1. Mol Pharmacol.

[26] Meotti FC, Lemos de Andrade E, Calixto JB (2014). TRP modulation by natural compounds. Handb Exp Pharmacol.

[27] Kobayashi Y (2003). The nociceptive and anti-nociceptive effects of evodiamine from fruits of *Evodia rutaecarpa* in mice. Planta Med.

[28] Anand P, Bley K (2011). Topical capsaicin for pain management: therapeutic potential and mechanisms of action of the new high-concentration capsaicin 8% patch. Br J Anaesth.

[29] Webster LR, Peppin JF, Murphy FT, Tobias JK, Vanhove GF (2012). Tolerability of NGX-4010, a capsacin 8% patch, in conjunction with three topical anesthetic formulations for the treatment of neuropathic pain. J Pain Res.

[30] Mainka T, Malewicz NM, Baron R, Enax-Krumova EK, Treede RD, Maier C (2016). Presence of hyperalgesia predicts analgesic efficacy of topically applied capsaicin 8% in patients with peripheral neuropathic pain. Eur J Pain.

[31] Touska F, Marsakova L, Teisinger J, Vlachova V (2011). A “cute” desensitization of TRPV1. Curr Pharm Biotechnol.

[32] Koplas PA, Rosenberg RL, Oxford GS (1997). The role of calcium in the desensitization of capsaicin responses in rat dorsal root ganglion neurons. J Neurosci.

[33] Mandadi S, Numazaki M, Tominaga M, Bhat MB, Armati PJ, Roufogalis BD (2004). Activation of protein kinase C reverses capsaicin-induced calcium-dependent desensitization of TRPV1 ion channels. Cell Calcium.

[34] Mohapatra DP, Nau C (2005). Regulation of Ca^2+^-dependent desensitization in the vanilloid receptor TRPV1 by calcineurin and cAMP-dependent protein kinase. J Biol Chem.

[35] Numazaki M, Tominaga T, Takeuchi K, Murayama N, Toyooka H, Tominaga M (2003). Structural determinant of TRPV1 desensitization interacts with calmodulin. Proc Natl Acad Sci USA.

[36] Rosenbaum T, Gordon-Shaag A, Munari M, Gordon SE (2004). Ca^2+^/calmodulin modulates TRPV1 activation by capsaicin. J Gen Physiol.

[37] Lishko PV, Procko E, Jin X, Phelps CB, Gaudet R (2007). The ankyrin repeats of TRPV1 bind multiple ligands and modulate channel sensitivity. Neuron.

[38] Sanz-Salvador L, Andrés-Borderia A, Ferrer-Montiel A, Planells-Cases R (2012). Agonist- and Ca^2+^-dependent desensitization of TRPV1 channel targets the receptor to lysosomes for degradation. J Biol Chem.

[39] Kennedy WR, Vanhove GF, Lu SP, Tobias J, Bley KR, Walk D, Wendelschafer-Crabb G, Simone DA, Selim MM (2010). A randomized, controlled, open-label study of the long-term effects of NGX-4010, a high-concentration capsaicin patch, on epidermal nerve fiber density and sensory function in healthy volunteers. J Pain.

[40] Wei P, Huo HL, Ma QH, Li HC, Xing XF, Tan XM, Luo JB (2014). Pharmacokinetic comparisons of five ephedrine alkaloids following oral administration of four different Mahuang-Guizhi herb-pair aqueous extracts ratios in rats. J Ethnopharmacol.

[41] Kaneko Y, Szallasi A (2014). Transient receptor potential (TRP) channels: a clinical perspective. Br J Pharmacol.

[42] Gavva NR, Treanor JJ, Garami A, Fang L, Surapaneni S, Akrami A, Alvarez F, Bak A, Darling M, Gore A, Jang GR, Kesslak JP, Ni L, Norman MH, Palluconi G, Rose MJ, Salfi M, Tan E, Romanovsky AA, Banfield C, Davar G (2008). Pharmacological blockade of the vanilloid receptor TRPV1 elicits marked hyperthermia in humans. Pain.

[43] Rowbotham MC, Nothaft W, Duan WR, Wang Y, Faltynek C, McGaraughty S, Chu KL, Svensson P (2011). Oral and cutaneous thermosensory profile of selective TRPV1 inhibition by ABT-102 in a randomized healthy volunteer trial. Pain.

