# Correction to: Ephedra Herb extract activates/desensitizes transient receptor potential vanilloid 1 and reduces capsaicin-induced pain

**DOI:** 10.1007/s11418-018-1196-8

**Published:** 2018-03-01

**Authors:** Shunsuke Nakamori, Jun Takahashi, Sumiko Hyuga, Toshiko Tanaka-Kagawa, Hideto Jinno, Masashi Hyuga, Takashi Hakamatsuka, Hiroshi Odaguchi, Yukihiro Goda, Toshihiko Hanawa, Yoshinori Kobayashi

**Affiliations:** 1 Department of Pharmacognosy, School of Pharmacy, Kitasato University, 5-9-1 Shirokane, Minato-ku, Tokyo, 108-8641 Japan; 2 Oriental Medicine Research Center, Kitasato University, 5-9-1, Shirokane, Minato-ku, Tokyo, 108-8642 Japan; 3 Department of Biochemical Toxicology, Yokohama University of Pharmacy, 601 Matano-cho, Totsuka-ku, Yokohama, 245-0066 Japan; 4 Faculty of Pharmacy, Meijo University, 150 Yagotoyama, Tempaku-ku, Nagoya, 468-8503 Japan; 5 National Institute of Health Sciences, 1-18-1 Kamiyoga, Setagaya-ku, Tokyo, 158-8501 Japan

## Correction to: J Nat Med (2017) 71:105–113 10.1007/s11418-016-1034-9

The article Ephedra Herb extract activates/desensitizes transient receptor potential vanilloid 1 and reduces capsaicin-induced pain, written by Shunsuke Nakamori, Jun Takahashi, Sumiko Hyuga, Toshiko Tanaka-Kagawa, Hideto Jinno, Masashi Hyuga, Takashi Hakamatsuka, Hiroshi Odaguchi, Yukihiro Goda, Toshihiko Hanawa and Yoshinori Kobayashi, was originally published Online First without open access. After publication in volume 71, issue 1, page 105–113 the author decided to opt for Open Choice and to make the article an open access publication. Therefore, the copyright of the article has been changed to © The Author(s) 2018 and the article is forthwith distributed under the terms of the Creative Commons Attribution 4.0 International License (http://creativecommons.org/licenses/by/4.0/), which permits use, duplication, adaptation, distribution and reproduction in any medium or format, as long as you give appropriate credit to the original author(s) and the source, provide a link to the Creative Commons license, and indicate if changes were made.

