# Supplementary figures and images for: Ephedra Herb extract activates/desensitizes transient receptor potential vanilloid 1 and reduces capsaicin-induced pain
Source: J Nat Med. 2016 Sep 8;71(1):105–13. doi: 10.1007/s11418-016-1034-9 (PMC5897484; doi:10.1007/s11418-016-1034-9)

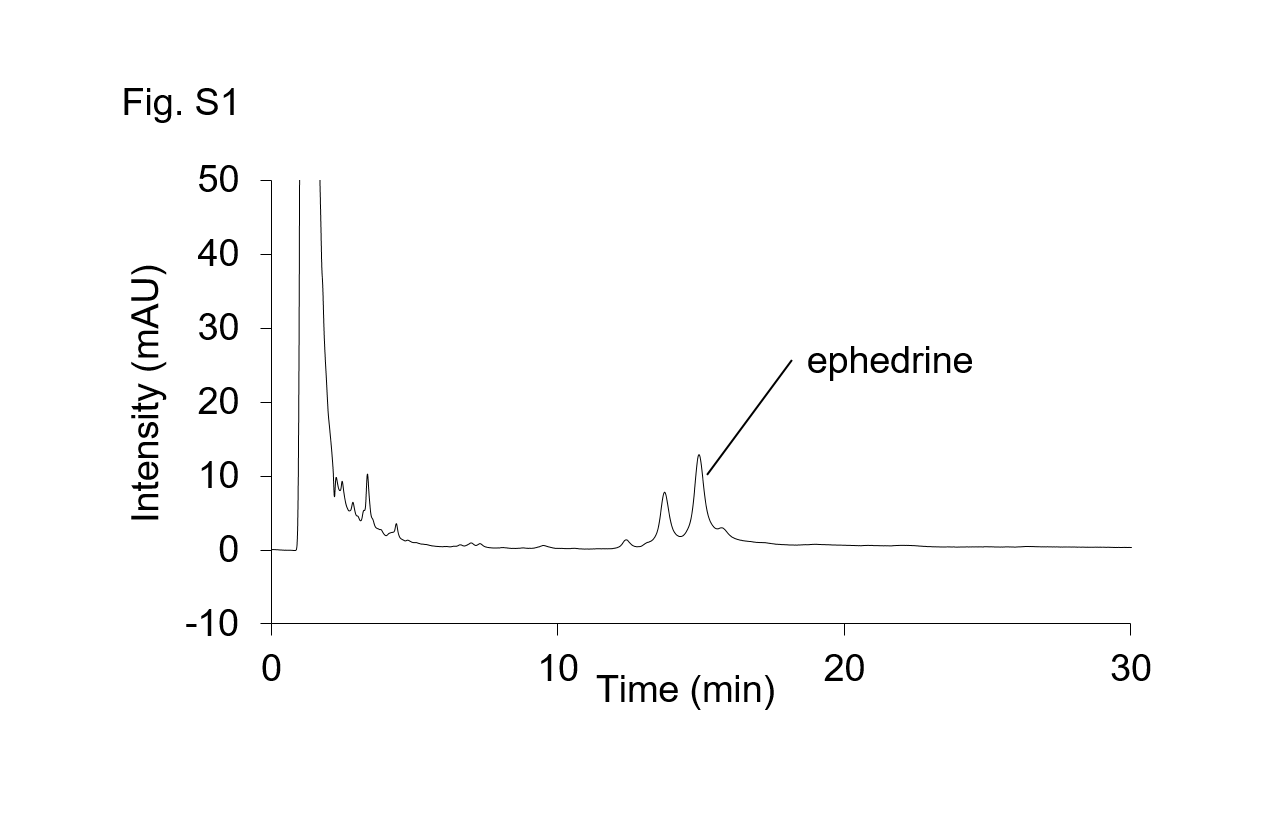

Supplement: Supplementary file 1 — Fig. S1 Chromatogram of EHE obtained using an HPLC system (Shimadzu, Kyoto, Japan) consisting of an SIL-20A auto-injector, SPD-M20A photodiode array detector, LC-20AD pump, DGU-20A3 degasser, and CBM-20A communications bus module. Separations were carried out with an YMC-Triart C18 plus column (5 µm particle size, 4.6 mm (inner diameter) × 150 mm; YMC Co., Ltd, Kyoto, Japan). The mobile phase was a mixture of water, acetonitrile, and phosphoric acid (650:350:1, v/v/v) containing 0.5 % SDS delivered at a flow rate of 1 ml/min. The column temperature was maintained at 40 °C with a CTO-20A column oven (Shimadzu). The detection wavelength was set at 210 nm for quantitative determination. The test samples were resolved by methanol and injected (10 µl injection volume) by an auto-injector. (TIFF 112 kb) [file 11418_2016_1034_MOESM1_ESM.tif]
